# Computational analysis of SARS-CoV-2, SARS-CoV, and MERS-CoV genome using MEGA

**DOI:** 10.5808/GI.2020.18.3.e30

**Published:** 2020-09-24

**Authors:** Vipan Kumar Sohpal

**Affiliations:** Department of Chemical & Bio Engineering, Beant College of Engineering & Technology, Gurdaspur 143521, India

**Keywords:** Middle East respiratory syndrome, Molecular Evolutionary Genetic Analysis, National Center for Biotechnology Information, SARS-CoV, SARS-CoV-2

## Abstract

The novel coronavirus pandemic that has originated from China and spread throughout the world in three months. Genome of severe acute respiratory syndrome coronavirus 2 (SARS-CoV-2) predecessor, severe acute respiratory syndrome coronavirus (SARS-CoV) and Middle East respiratory syndrome coronavirus (MERS-CoV) play an important role in understanding the concept of genetic variation. In this paper, the genomic data accessed from National Center for Biotechnology Information (NCBI) through Molecular Evolutionary Genetic Analysis (MEGA) for statistical analysis. Firstly, the Bayesian information criterion (BIC) and Akaike information criterion (AICc) are used to evaluate the best substitution pattern. Secondly, the maximum likelihood method used to estimate of transition/transversions (R) through Kimura-2, Tamura-3, Hasegawa-Kishino-Yano, and Tamura-Nei nucleotide substitutions model. Thirdly and finally nucleotide frequencies computed based on genomic data of NCBI. The results indicate that general times reversible model has the lowest BIC and AICc score 347,394 and 347,287, respectively. The transition/transversions bias for nucleotide substitutions models varies from 0.56 to 0.59 in MEGA output. The average nitrogenous bases frequency of U, C, A, and G are 31.74, 19.48, 28.04, and 20.74, respectively in percentages. Overall the genomic data analysis of SARS-CoV-2, SARS-CoV, and MERS-CoV highlights the close genetic relationship.

## Introduction

Coronaviruses (CoVs) usually influence the respiratory tract of mammals that lead to mild to severe respiratory tract infections [1]. In the past two decades, two highly pathogenic human CoVs including severe acute respiratory syndrome coronavirus (SARS-CoV) and Middle East respiratory syndrome coronavirus (MERS-CoV), emerging from animal reservoirs, have led to global epidemics with high morbidity and mortality [2]. According to the World Health Organization (WHO), as of April 2020 CoV has had a total of 2,269,630 diagnosed cases causing 155,205 deaths, throughout the world [3]. SARS-CoV-2 has a similar incubation phase and a relatively lower fatality rate than SARS-CoV or MERS-CoV, but it is estimated that the reproductive number of SARS-CoV-2 is higher than that of SARS-CoV [4]. Whole-genome analysis revealed that SARS-CoV-2 used mutations and recombination as crucial strategies in different genomic regions to become a novel infectious agent [5].

Generally, the rates of nucleotide substitution of RNA viruses are faster and are this rapid evolution is mainly shaped by natural selection [6]. Numerous substitution models are time-reversible and, the model does not care which sequence is the ancestor and which is the descendant so long as all other parameters are held constant. Generalized time reversible (GTR) is the most general neutral, independent, finite-sites, time-reversible model possible [7]. On other hand, there are 203 possible ways that the exchangeability parameters can be restricted to form sub-models of GTR, ranging from the JC69 and F81 models (where all exchangeability parameters are equal) to the SYM model and the full GTR model (where all exchangeability parameters are free) [8-10]. The Jukes-Cantor (JC or JC69) model assumes equal transition rates as well as equal equilibrium frequencies for all bases and it is the simplest sub-model of the GTR model [11]. Kimura 2 (K2) parameters model and three parameters model are conserved the strong/weak properties of nucleotides [12,13]. F81 and HKY (five parameters) models in which the substitution rate are corresponds to the equilibrium frequency of the target nucleotide [14]. Bayesian information criterion (BIC) and Akaike information criterion (AICc) statistical models is important tool in analysis biological data [15]. In addition to that there are several methods for estimating substitution rates from genome sequence data [16].

As an emerging virus, limited information is available to depict the genetic diversity and nucleotide substitution and rate. Hence the purpose of the present work is to assess the genomic relationship on the basis of statistical techniques between MERS-CoV, SARS-CoV, and SARS-CoV-2 with an objective to (1) maximized value of likelihood function of nucleotide substitution models, (2) transition/transversion bias and frequencies computation using maximum likelihood (ML) technique, (3) analyze the probability rate of substitution using ML. It is assumed MERS-CoV, SARS-CoV, and SARS-CoV-2 belong to same phylogeny due to respiratory syndrome, but the present manuscript on the basis of genomic data able to depict the biological relationship. The comparison of the genomic data with various substitutions techniques is presented to analyze the relationship.

## Methods

The genomic data for substitution analysis of SARS-CoV-2 (NC_045512.2), MERS-CoV (NC_019843.3), and SARS-CoV (FJ588686.1) viruses were obtained from the National Center of Biotechnology Information (NCBI) using Molecular Evolutionary Genetic Analysis (MEGA) [17] bioinformatics tool. Filtration of NCBI database through general nucleotide collection used of Megablast to optimize highly similar sequences. Filter and Mask of Blast used for filtration of data: (a) Filter (low complexity region filter) and (b) Mask (Query masked on using to scan database). ML statistical method used to compute BIC score and AICc value of 24 different nucleotide substitution models. Mathematically BIC is function of *f (n, k, L)*, AIC *f (k, L)*, *AICc f (AIC, k, n)* is as mentioned in Eqs. (1), (2), and (3).

(1) BIC=ln(n)k-21n(L)

(2) AIC=2k-21n(L)

(3) $$AIC_{c}=AIC+\frac{2k^{2}+2k}{n-k-1}$$

*L* = the maximized value of likelihood function of model M, *n* = number of data point, *K* = number of parameters estimated by model. Frequencies and transition/transversion bias 24 different nucleotide substitution models also evaluated. Simulate the biological data to estimate the probability rate of substitution (*r*) using ML method for different nucleotide substitution models. Similarly, database used to assess the nucleotide base frequencies for each sequence as well as an overall average to assess the extent of relation.

## Results and Discussion

### ML of different nucleotide substitution models

BIC and AICc are the most important parameters for statistical analysis of ML to analyze the biological data. Both the BIC and AICc used to evaluate the best model among a finite set of models with penalty parameters. BIC based, on the likelihood function and AICc estimator of out-of-sample prediction error.

**Fig. 1.**
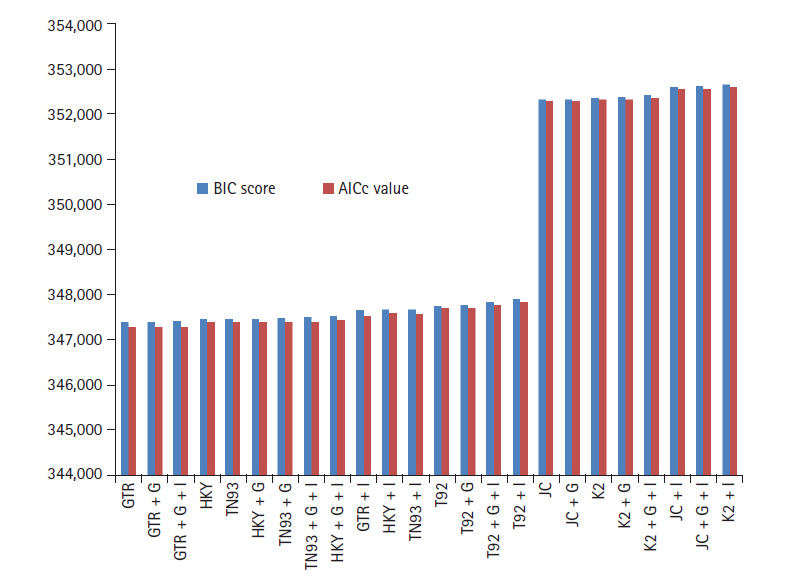
Bayesian information criterion (BIC) score and Akaike information criterion (AICc) value for maximum likelihood fits of 24 different nucleotide substitution models.

GTR model have lowest BIC and AICc score 347,395, 347,288 computed using MEGA with *K* = 11 shown in Fig. 1. In addition, rate of variation across sites (+G), the GTR + G model show BIC and AIC score slightly increase with respect to GTR. On further addition, a proportion of invariable sites (+I) and/or rate of variation across sites (+G), GTR + G + I model indicates 0.0072% elevation in BIC score and 0.00144% go up in AIC (*K* = 13). HKY model (*K* = 7) having lowest value for BIC 347473, AICc 347405, but higher than most appropriate GTR model. Similarly HKY + I + G model (*K* = 9) simulated result shows the score get higher with respect to base model. Both the model JC + G + I and K2 + I (*K* = 5) boast BIC and AICc criterion score highest. The deviation between GTR and K2 + I models is for BIC, AICc scores 1.49% and 1.50%, respectively.

It indicates ML method accurately fits of 24 different nucleotide substitution models for biological data of SARS-CoV-2, MERS-CoV, and SARS-CoV under neutral evolution. As per information theory, lowest BIC score preferred due to Bayesian probability and inference, while highest score criteria opted for AICc based on frequentist-based inference. Simulative investigation results reveal that differences between lowest and highest scores are around 1.5%, virtue of that SARS-CoV-2, MERS-CoV, and SARS-CoV data best fitted through GTR model. The corrected AIC model gives better results as compare to AIC value as correlated in Eq. (3).

**Fig. 2.**
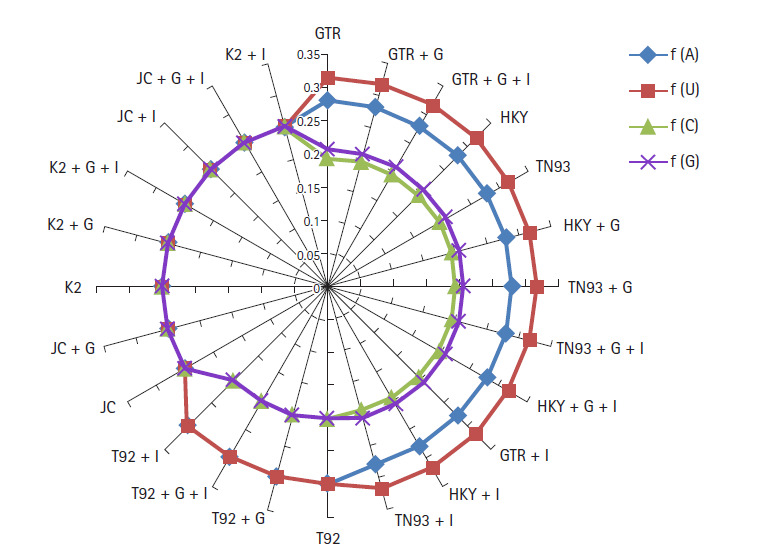
Frequencies of severe acute respiratory syndrome coronavirus 2 (SARS-CoV-2), Middle East respiratory syndrome coronavirus, SARS-CoV genome using nucleotide substitution with maximum likelihood approach.

**Fig. 3.**
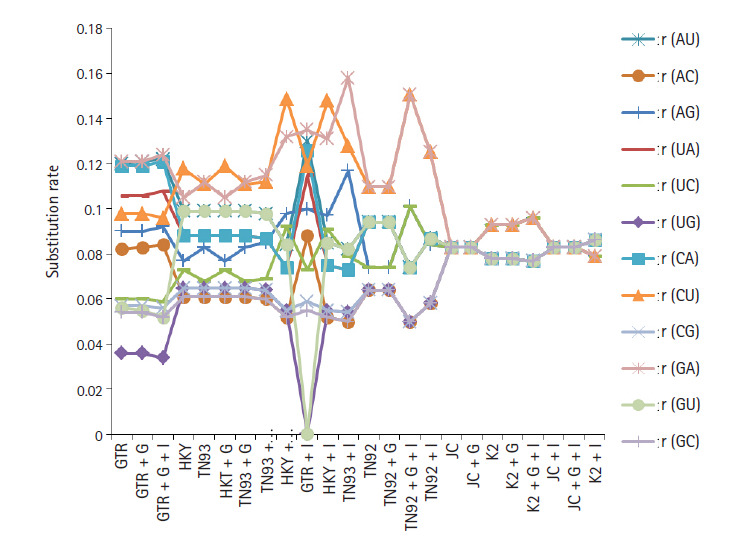
Substitution rate for severe acute respiratory syndrome coronavirus 2 (SARS-CoV-2), Middle East respiratory syndrome coronavirus, SARS-CoV genome using maximum likelihood for different nucleotide substitution models.

Nucleotide frequencies (*f*) and rates of base substitutions rate (*r*) are also key factor to justify best nucleotide substitution model using ML technique. The nucleotide frequencies predicted for GTR model are A = 0.28, U = 0.317, C = 0.195 and G = 0.207 of biological data of SARS-CoV-2, SARS-CoV, and MERS-CoV. The frequencies of nitrogenous base remain constant for first 12 models from GTR, GTR + G,GTR + G + I, HKY, TN93, HKY + G, TN93 + G, TN93 + G + I, HKY + G + I, GTR + I, HKY + I to TN93 + I. The nucleotide frequencies for T92, T92 + G, T92 + G + I, T92 + I models are (A = 0.299, U = 0.299, C = 0.201, G = 0.201) remain steady, but varied from prior methods. JC, JC + G, K2, K2 + G, K2 + G + I, JC + I, JC + G + I, K2 + I models replicated the same frequency at the rate 0.25 for all nitrogenous base as revealed in Fig 2. Base substitution rates are also dependent on nucleotide substitutions models, in GTR model r(AU), r(UA), r(CA), r(GA) substitutions are dominated. Fig. 3 replicate the min and max rate of rates of base substitutions irrespective of models are as follow, r(AU 0.077, 0.122), r(AC 0.05, 0.084), r(AG 0.77, 0.107), r(UA 0.074, 0.115), r(UC 0.06, 0.101), r(UG 0, 0.086), r(CA 0.073, 0.126), r(CU 0.079, 0.119), r(CG 0.05, 0.124), r(GA 0.079, 0.132), r(GU 0, 0.099), and r(GC 0.05, 0.086).

It has been observed that under the model of uniform substitution among site REV, TN93 HKY, the frequency parameters are free to exchangeability, while JC and K2 models have frequencies at uniform rate 1/4. Virtue of these statistical parameters, the models GTR, GTR + G, GTR + G + I, HKY, TN93, HKY + G, TN93 + G, TN93 + G + I, HKY + G + I, GTR + I, HKY + I, and TN93 + I shows similar results in term nucleotide frequencies. JC and K2 model rely on different frequency parameter, due to that JC, JC + G, K2, K2 + G, K2 + G + I, JC + I, JC + G + I, and K2 + I models replicate the result same mode. The estimates of transitional and transversional of substitution rates are of 1st + 2nd + 3rd position data using simulation of data. Fig. 3 confirms that the number of transversional are larger than the number of transitions. In broad, the transitional/transversional varies from 0.57 (GTR model) to 0.89 (T92 + G + I), higher values indicate proportion of invariable sites (+I) and/or rate of variation across sites (+G) are more dominating in T92 model for SARS-CoV-2, SARS-CoV, and MERS-CoV biological sequence.

### ML to estimate of substitution matrix and transition/transversion bias

Probability rate of substitution (*R*) using ML depends upon the base frequency parameters and nucleotide substitution models. Base frequency parameters Π_A_ = Π_C_ = Π_T_ = Π_U_ = 1/4 for JC and K2 models and for GTR, HKY, TN93, T3 models have all Πi free to exchange. Six different nucleotide substitution models were simulated for biological sequence data of SARS-CoV, MERS-CoV, and SARS-CoV-2.

JC substitution model shows the transitional and transversionsal substitutions rate 8.33, while transitional substitutions for all base are 9.32 and transversionsal substitutions is equal to 7.84 for K2 parameter model. In general, HKY, TN93 models having transitional substitutions are more dominating in C-U and transitional substitution G-U and A-U. GTR and T3 parameter models resultant of higher transition substitution for A-G, 11.24 and 12.13, respectively. The lowest value of transition in GTR and T3 models also lies for same base (C-U). The highest probabilities of transversional substitutions (A-U) are the models are 9.93 and 12.05 as shown in Table 1. In all models except than JC and K2, the lowest transitional substitutions observed C-U base pair. Overall transitional substitutions have higher hand as compare transversional substitutions in all models.

**Fig. 4.**
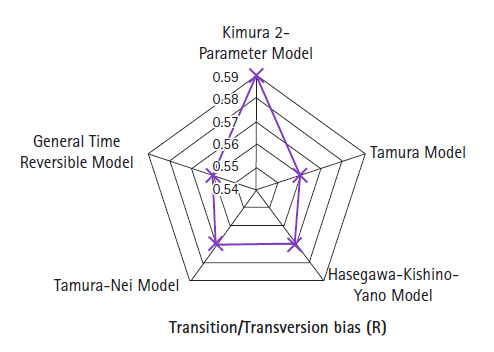
Transition/transversion bias for different nucleotide substitution models.

The estimated transition/transversion bias is 0.59 for K2-parameter model with codon positions included 1st + 2nd + 3rd + Noncoding that is not translated into a protein. There are a total of 43,053 positions in the final dataset. The transition/transversion bias for T93 and GTR equal to 0.56, while HKY and T3 parameter have transition/transversion bias is 0.57 as revealed in Fig. 4. The variations in the entire model are from 0.56 to 0.59, and overall consistent value for transition/transversion bias.

JC and K2 models belong to one class of base frequency parameters, virtue of that JC model demonstrates equal rate of transition/transversion bias. K2 model shows constant rate of transition 9.32 and transversional 7.84 substitution biases. On the other hand, T93, T3, HKY, and GTR model exchangeability are free, due to that transitional and transversionsal substitutions rate are different. Transi­tion/transversion bias is approximately 0.5 when that indicates no bias towards either transitional or transversional substitution because two kinds of substitution are equally probable, there are twice as many possible transversions as transitions.

**Fig. 5.**
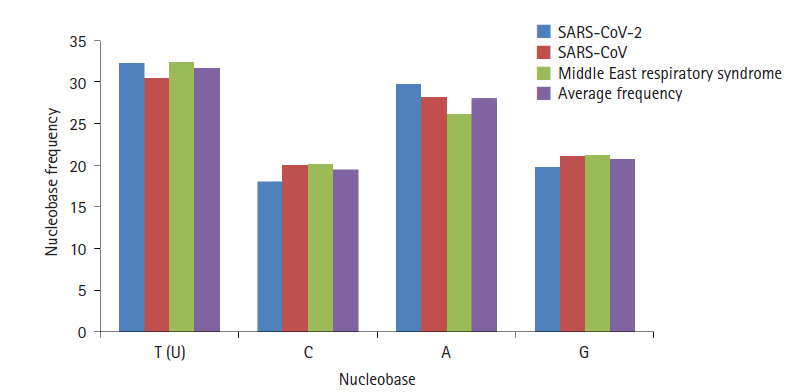
Nucleobase frequencies for severe acute respiratory syndrome coronavirus 2 (SARS-CoV-2), SARS-CoV, Middle East respiratory syndrome coronavirus (MERS-CoV) and average frequency against respective nucleobase.

SARS-CoV-2 nucleobase has higher frequency of T as compared to SARS-CoV, and approximately equal to MERS-CoV. Cytosine frequency of SARS-CoV-2 is less than both the biological sequences of SARS-CoV and MERS-CoV as shown in Fig 5. The variation in cytosine base is around 9.6% with respect to SARS-CoV. The adenine nucleobase frequency is 29.896 of SARS-CoV-2 much higher than MERS-CoV and 5.75% modified from SARS-CoV. On the other hand, guanine frequency for current SARS-CoV-2 is much lesser than both the SARS-CoV and MERS-CoV. The average frequency of SARS-CoV-2, SARS-CoV, and MERS-CoV for U, C, A, and G are 31.74012, 19.48521, 28.04331, and 20.73135, respectively. Close value of nucleobase frequency (SARS-CoV-2, MERS-CoV, and SARS-CoV) reflects that SARS-CoV-2 is modified from previous respiratory syndrome virus.

### Conclusions

On the basis of BIC and AICc score, it concluded that GTR model is more accurate for genome analysis of SARS-CoV & MERS-CoV and CoV-2 under non-uniform rates of evolution and invariable (*+I*). 0.03% difference found in BIC and AIC score for GTR model at penalty parameter of 11 signified that SARS-CoV-2 is closely to SARS-CoV and MERS-CoV both virus strains. The base frequency all 24-substitution model except JC and K2 are same, due to free exchangeability, resultant of that JC and K2 parameter observations trends are different from other substitution models. The results also indicate the close proximity of SARS-CoV-2 to SARS-CoV and MERS-CoV probability rate of substitution confirmed transitional substitutions are more dominate in all genomic sequences (NC_045512.2, NC_019843.3, and FJ588686.1) because two out of three single nucleotide polymorphisms are transitions retain in SARS-CoV, MERS-CoV, and SARS-CoV-2. Low frequency of nucleotide (0–0.35) and substitution rate (0–0.18) in all nucleotide substitution models support the result of closeness among the virus strain. 1st + 2nd + 3rd + noncoding simulated result for transi­tion/transversion bias reflected the positive evolution that indicates towards of nonsynonymous substitutions. The outcome of A-T (62.14%) and G-C (37.86%) nucleobase frequencies for SARS-CoV-2 evidence that variation in genome with respect SARS-CoV & MERS-CoV. The G-C frequencies are 5.86% elevated in SARS-CoV & MERS-CoV and A-T frequencies are 5.86% upward for SARS-CoV-2. Closer the nucleobase frequency also supports and affirms SARS-CoV-2 is closer resemblance of SARS-CoV and MERS-CoV.

## Figures and Tables

**Table 1.** Probability rate of substitution (R) using maximum likelihood statistical method

	Substitution rate			
	A	T/U	C	G
Juke Cantor Model				
A	-	*8.33*	*8.33*	**8.33**
T/U	*8.33*	-	**8.33**	*8.33*
C	*8.33*	**8.33**	-	*8.33*
G	**8.33**	*8.33*	*8.33*	-
Tamura Model				
A	-	*9.45*	*6.36*	**7.4**
T/U	*9.45*	-	**7.4**	*6.36*
C	*9.45*	**11**	-	*6.36*
G	**11**	*9.45*	*6.36*	-
Tamura-Nei Model				
A	-	*9.93*	*6.1*	**8.31**
T/U	*8.78*	-	**6.79**	*6.49*
C	*8.78*	**11.06**	-	*6.49*
G	**11.24**	*9.93*	*6.1*	-
Kimura-2 Parameter Model				
A	-	*7.84*	*7.84*	**9.32**
T/U	*7.84*	-	**9.32**	*7.84*
C	*7.84*	**9.32**	-	*7.84*
G	**9.32**	*7.84*	*7.84*	-
Hasegawa-Kishino-Yano Model				
A	-	*9.95*	*6.11*	**7.74**
T/U	*8.79*	-	**7.27**	*6.5*
C	*8.79*	**11.84**	-	*6.5*
G	**10.46**	*9.95*	*6.11*	-
General Time Reversible Model				
A	-	*12.05*	*8.25*	**8.97**
T/U	*10.64*	-	**6**	*3.64*
C	*11.87*	**9.78**	-	*5.72*
G	**12.13**	*5.57*	*5.38*	-

Transitional substitutions are shown in bold and transversionsal substitutions are shown in italics.
